# *In planta* Production and Validation of Neuraminidase Derived from Genotype 4 Reassortant Eurasian Avian-like H1N1 Virus as a Vaccine Candidate

**DOI:** 10.3390/plants11212984

**Published:** 2022-11-04

**Authors:** Da Been Kim, Sun Min Lee, Kyoung Rok Geem, Jitae Kim, Eui Ho Kim, Dong Wook Lee

**Affiliations:** 1 Department of Integrative Food, Bioscience and Biotechnology, Chonnam National University, Gwangju 61186, Korea; 2 Viral Immunology Laboratory, Institut Pasteur Korea, Seongnam 13488, Korea; 3 Department of Bioenergy Science and Technology, Chonnam National University, Gwangju 61186, Korea; 4 Bio-Energy Research Center, Chonnam National University, Gwangju 61186, Korea

**Keywords:** G4 Eurasian avian-like H1N1 virus, neuraminidase, vaccine, molecular farming, immune response

## Abstract

Influenza viruses are a major public health threat that causes repetitive outbreaks. In recent years, genotype 4 (G4) reassortant Eurasian avian-like (EA) H1N1 (G4 EA H1N1) has garnered attention as a potential novel pandemic strain. The necessity of developing vaccines against G4 EA H1N1 is growing because of the increasing cases of human infection and the low cross-reactivity of the strain with current immunity. In this study, we produced a G4 EA H1N1-derived neuraminidase (G4NA) as a vaccine candidate in *Nicotiana benthamiana*. The expressed G4NA was designed to be accumulated in the endoplasmic reticulum (ER). The M-domain of the human receptor-type tyrosine-protein phosphatase C was incorporated into the expression cassette to enhance the translation of G4NA. In addition, the family 3 cellulose-binding module and *Brachypodium distachyon* small ubiquitin-like modifier sequences were used to enable the cost-effective purification and removal of unnecessary domains after purification, respectively. The G4NA produced in plants displayed high solubility and assembled as a tetramer, which is required for the efficacy of an NA-based vaccine. In a mouse immunization model, the G4NA produced in plants could induce significant humoral immune responses. The plant-produced G4NA also stimulated antigen-specific CD4 T cell activation. These G4NA vaccine-induced immune responses were intensified by the administration of the antigen with a vaccine adjuvant. These results suggest that G4NA produced in plants has great potential as a vaccine candidate against G4 EA H1N1.

## 1. Introduction

Influenza A virus, including the subtype H1N1, has been a threat to public health by causing flu endemics and pandemics throughout history. Influenza A virus is classified into subtypes based on the type of two antigenic surface proteins, hemagglutinin (HA) and neuraminidase (NA). Among the subtypes, A(H1N1), A(H3N2), and A(H5N1) are the only ones that are currently circulating in humans. Current H1N1 infections in humans are mostly related to A(H1N1)pdm09, therefore, the seasonal flu vaccines contain HA derived from the A(H1N1)pdm09 for protection against it [1]. However, recently, genotype 4 (G4) reassortant Eurasian avian-like (EA) H1N1 (G4 EA H1N1) has emerged as a reassortant that requires monitoring. G4 EA H1N1 is a predominant genotype among reassortants between the classical swine H1N1 and human A(H1N1)pdm09 [2]. One study demonstrated that G4 EA H1N1 binds to human-like Saα2,6Gal receptor, which is an important factor for human cell infection, and can replicate in human airway epithelial cells [3]. This is alarming, as these reassortants show low cross-reactivity to pre-existing immunity obtained by current influenza vaccines [3].

To develop influenza vaccines, the surface proteins of the influenza virus have been used as dominant targets for antigens. There are two major glycoproteins on the surface of the influenza virus: HA and NA. These proteins are essential in the viral infection process. HA is more abundant on the surface of the influenza virus than NA and critically contributes to virus entry by directly binding to sialic acids present on the host cell surface. On the other hand, NA cleaves the sialic acids, enabling the release of the virus from host cells. Currently licensed influenza vaccines are majorly formulated and optimized based on the HA antigen [4], whereas NA is not utilized in these vaccines. However, several studies have revealed the importance of NA as a vaccine antigen [5,6]. Importantly, anti-NA antibodies elicited a protective effect against the influenza virus [7,8]. The NA antigen evolves independently of HA [9,10], and NA induces heterologous immunity. For example, cross-protective anti-NA antibodies were generated after a seasonal vaccination with a single influenza viral strain, A/California/07/2009 (subtype A (H1N1) pdm09) [11]. Due to these beneficial characteristics, NA has been suggested as a good antigen candidate to improve the breadth of vaccine efficacy and is applicable to vaccine design [6].

The plant expression system has recently drawn attention to the production of pharmaceutical and non-pharmaceutical proteins. In comparison to the mammalian or bacterial expression systems, protein expression in plants is much safer and more cost-effective. Although the protein yields of plant expression systems are relatively low, the process of growing plants is highly scalable. In addition, like other eukaryotic cells, plant cells are equipped with the mechanisms for posttranslational modifications, such as glycosylation in the ER, which is sometimes critical for the solubility and functionality of expressed proteins [12,13,14,15]. Moreover, several studies indicated that many proteins of different origins were fully functional when they were produced in plants [16,17,18,19,20]. Several pharmaceutical proteins such as vaccines, cytokines, and therapeutic antibodies have been successfully produced in plants such as tobacco, barley, carrot, potato, and maize [16,21,22,23,24,25,26,27]. Notably, transient expression systems using plant tissues rapidly provide large amounts of therapeutic proteins during epidemic or pandemic periods [28,29,30,31].

In this study, we attempted to produce G4 EA H1N1-derived neuraminidase (G4NA) in the leaf tissues of *N. benthamiana* as a vaccine candidate against G4 EA H1N1. The G4NA produced in the endoplasmic reticulum (ER) of *N. benthamiana* was highly soluble. In addition, the G4NA produced in plants exhibited tetramer formation, which is considered important for the immunogenicity of NA-based vaccines. Moreover, mice immunized with the antigen G4NA alone displayed moderate immunogenicity, including the production of antigen-specific antibody and T cell responses, which could be enhanced by an emulsion vaccine adjuvant.

## 2. Results and Discussion

### 2.1. Construct Design for the Expression of MCS-G4NA in Plants

**Figure 1:**
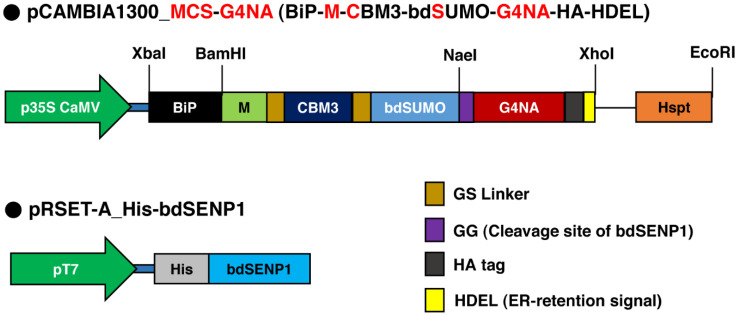
**Schematic representation of *MCS-G4NA* construct.** BiP, the signal sequence of BiP; M, the extracellular domain (amino acid residues 231–290) of human protein tyrosine phosphatase receptor type C; CBM3, the cellulose-binding module 3 of *Clostridium thermocellum*; bdSUMO, the SUMO domain of *Brachypodium distachyon*; and HDEL, ER retention signal.

The construct *MCS-G4NA* was incorporated into the pCAMBIA1300 binary vector (Figure 1). We used the signal sequence of binding immunoglobulin protein (BiP) for the cotranslational translocation of MCS-G4NA into the ER. A C-terminal HDEL sequence is an ER retention signal required for the accumulation of expressed MCS-G4NA in the ER. The M domain, which was derived from the human receptor-type tyrosine-protein phosphatase C, was incorporated because it increases the translational level of ER-localized proteins [32]. For the affinity purification of MCS-G4NA, we incorporated the domain CBM3, which effectively binds to MCC beads and provides cost-effective purification of various proteins from plants [17,18,19,20,33]. After purification with MCC beads, the domains upstream of G4NA were removed by His-bdSENP1 purified from *E. coli* BL21 (DE3) pLysS (Figure 1).

### 2.2. The MCS-G4NA Expressed in N. benthamiana Displays High Solubility and Stability

**Figure 2:**
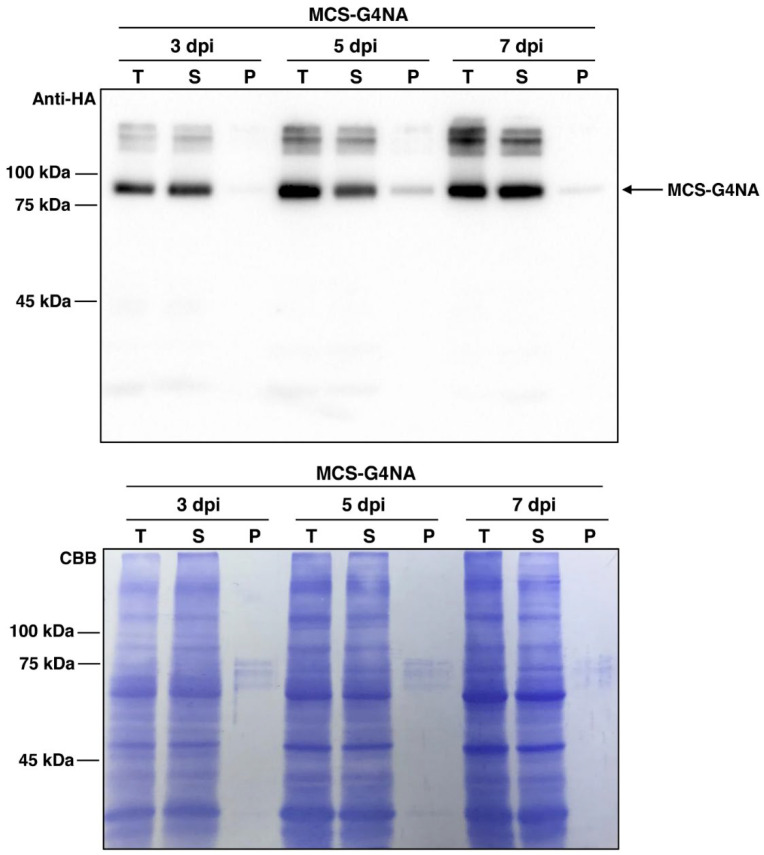
**Expression of *MCS-G4NA* in *N. benthamiana* leaves.** The leaf tissues of *N. benthamiana* were transformed with *Agrobacterium tumefaciens* cells harboring *MCS-G4NA*. *A. tumefaciens* cells harboring *P38* were co-infiltrated. The transformed leaves were harvested three, five, and seven days post-infiltration (dpi), followed by total protein extract preparation. Total protein extracts were subjected to centrifugation at 19,400× *g* for 10 min. Subsequently, the fractions soluble (S) from the supernatant and pellet (P) were collected separately for Western blot analysis using the anti-HA antibody. T, total fraction; S, soluble fraction; and P, pellet fraction. After Western blotting, the membrane was stained with Coomassie Brilliant Blue (CBB).

The construct *MCS-G4NA* was transformed into the leaves of *N. benthamiana* by syringe infiltration (Figure 2). At 3, 5, and 7 days post infiltration (dpi), the total protein extracts were prepared from transformed leaves and subjected to centrifugation at 19,400× *g* for 15 min at 4 °C. Then total, soluble, and pellet fractions were analyzed by anti-HA antibody (Figure 2). The MCS-G4NA whose predicted molecular weight is approximately 83 kilodaltons (kDa) was mainly detected in the soluble fraction (Figure 2). Moreover, the MCS-G4NA proteins were intact without proteolytic degradation. The protein bands above 83 kDa were considered aggregated or modified forms and were not further pursued in this study. These results indicate that the MCS-G4NA produced in *N. benthamiana* is soluble and stable.

### 2.3. Purification of G4NA from the Leaves of N. benthamiana

**Figure 3:**
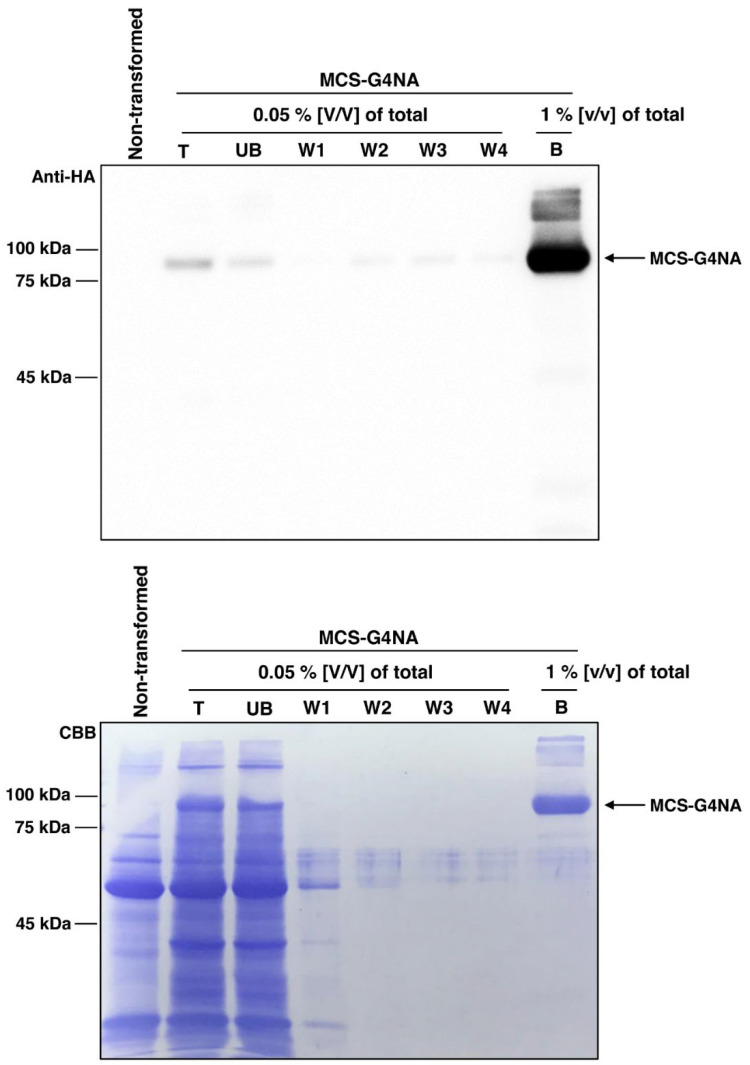
**Purification of MSC-G4NA from the *N. benthamiana* leaf extracts.** Total (T) protein extracts were centrifuged and the soluble protein fraction from the supernatant was incubated with MCC beads to purify MCS-G4NA. After centrifugation, the supernatant fraction was collected as the unbound (UB) fraction. Subsequently, the MCC beads were washed four times with a wash buffer (50 mM Tris-HCl and 150 mM NaCl; pH 7.5) (W1–W3). Finally, MCS-G4NA was eluted by boiling with a protein sample buffer. T, total protein extracts; UB, unbound fraction; and B, MCC bead-bound fraction. After Western blotting, the membrane was stained with Coomassie Brilliant Blue (CBB).

**Figure 4:**
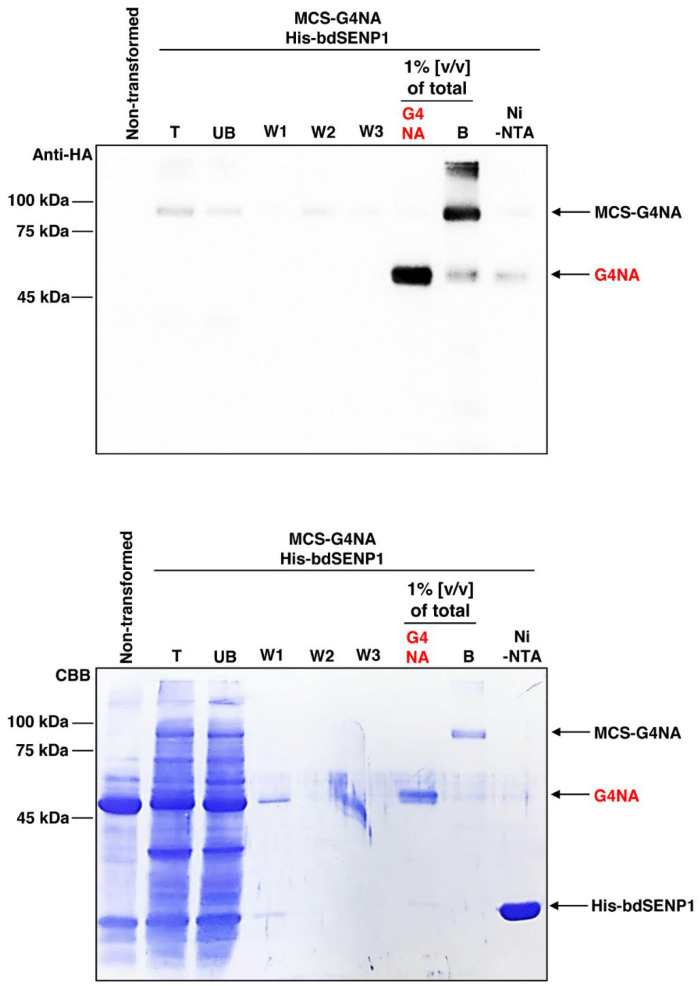
**bdSENP1-mediated removal of M, CBM3, and bdSUMO domains.** After purification of MCS-G4NA with MCC beads, the N-terminal upstream domains, which include CBM3 and bdSUMO, were removed by His-bdSENP1 protease, which was expressed in *E. coli* BL21 (DE3) pLysS. After the reaction, the supernatant was collected and passed through a Ni^2+^-NTA affinity column to remove His:bdSENP1. After Western blotting, the membrane was stained with Coomassie Brilliant Blue (CBB).

Next, we purified MCS-G3NA using MCC beads, which effectively bind to CBM3 (Figure 3) [17,18,33]. The protein samples in the soluble fraction in Figure 2 were incubated with MCC beads, followed by Western blotting with the anti-HA antibody. Expectedly, most of the expressed MCS-G4NA was immobilized with MCC beads, as MCS-G4NA is almost exclusively present in the soluble fraction (Figure 2) and MCS-G4NA was hardly detected in the unbound (UB) fraction (Figure 3). After purification, MCS-G4NA immobilized with MCC beads was treated with His-bdSENP1, which detects the bdSUMO domain and cleaves the di-glycine motif located at the C-terminus of the bdSUMO domain [33]. As shown in Figure 4, the CBM3 and bdSUMO domains upstream of G4NA were removed by His-bdSENP1. G4NA, which is used for vaccination, was released into the soluble fraction (Figure 4). After the on-bound cleavage reaction, His-bdSENP1 was removed using Ni-NTA beads (Figure 4). The protein yield of G4NA was estimated to be ~10 mg/kg fresh weight of *N. benthamiana* leaves. In this study, we took advantage of the syringe infiltration method. In the future, for an upscaled production of G4NA in *N. benthamiana* leaves, alternative methods need to be used for transformation, such as the vacuum infiltration method, which is more efficient than syringe infiltration and can quickly transform many plants [34], and the viral transfection of target genes [35,36].

### 2.4. G4NA Produced in N. benthamiana Is Present as a Tetramer

**Figure 5:**
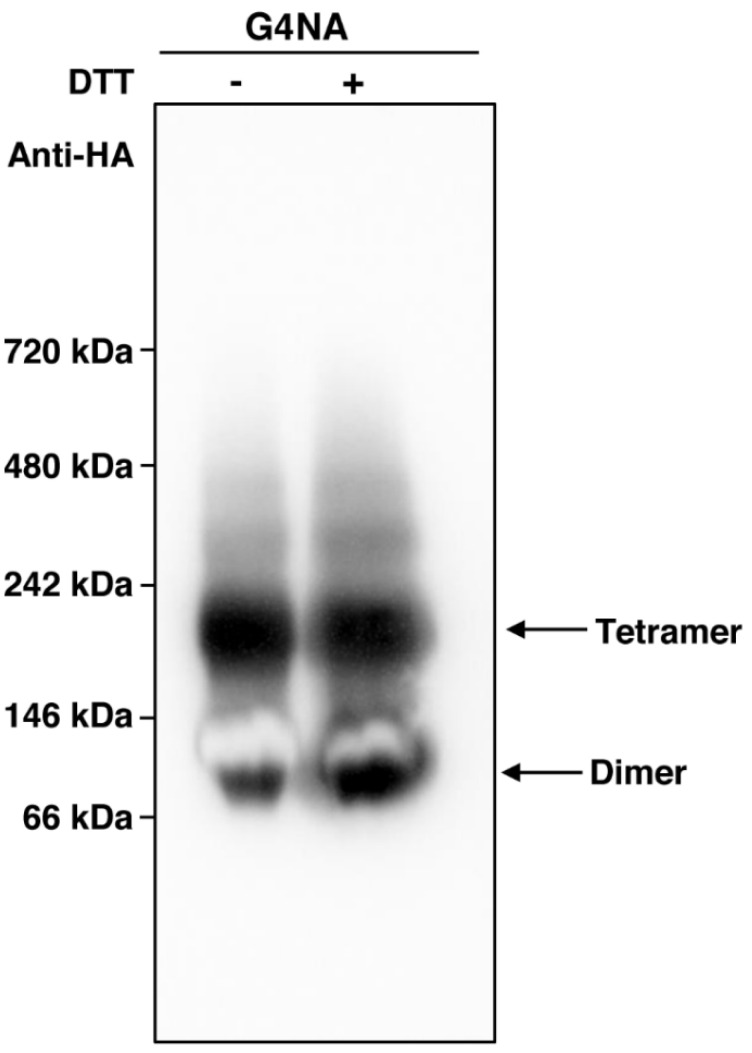
**Tetramer formation of G4NA produced in *N. benthamiana.*** After purification of G4NA in the absence (−) or presence (+) of DTT from the leaves of *N. benthamiana*, the purified G4NA was subject to BN-PAGE, followed by Western blotting with anti-HA antibody.

Previously, it has been suggested that the tetrameric form of NA enhances the protective immunity against A/PR8 (H1N1) infection in mice [37,38]. In this study, during the purification of G4NA, we used protein extraction buffer containing a reducing agent dithiothreitol (DTT) as described in Materials and Methods, which might affect tetramer formation of G4NA. To investigate whether G4NA produced in plants exhibits tetramer formation, we performed BN-PAGE using G4NA that was purified in the absence or presence of DTT (Figure 5). The expected size of G4NA monomer is approximately 48 kDa. As shown in Figure 5, the purified G4NA was mainly detected as about 200 kDa form, which corresponds to the size of G4NA tetramer, even if it was purified in the presence of DTT (Figure 5). This result indicates that G4NA produced in plants exhibits tetramer formation, which is required for the high efficacy of NA-based vaccines.

### 2.5. G4NA Produced in Plants Induces an Antigen-Specific Antibody Response

**Figure 6:**
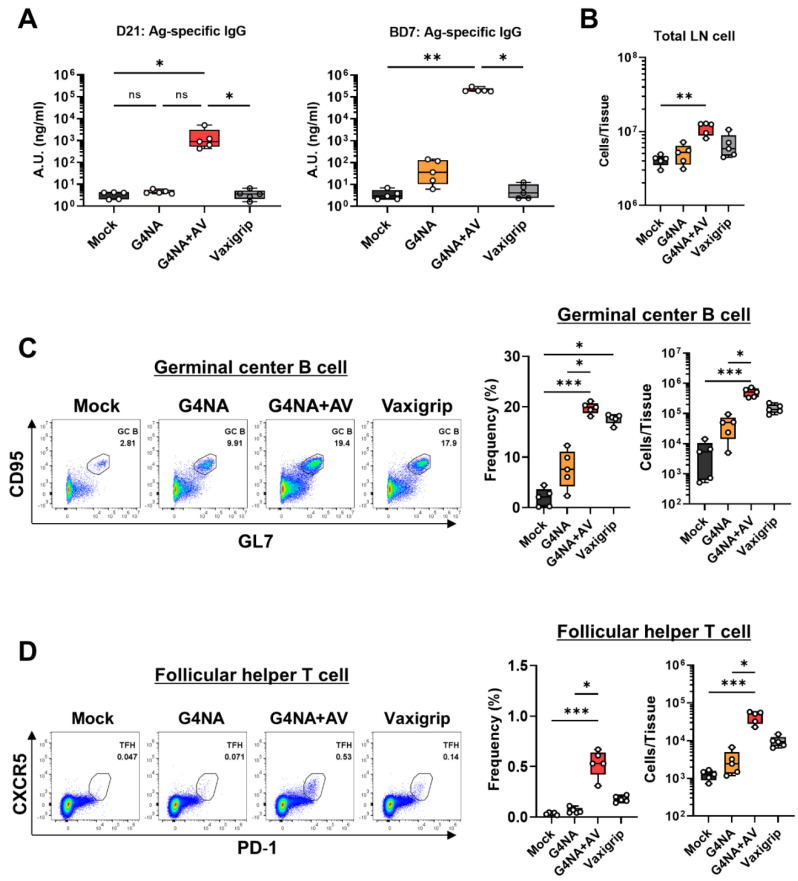
**G4NA induces antibody responses through the formation of germinal centers in lymph nodes after prime-boost vaccination with G4NA or G4NA plus AV or Vaxigrip Tetra**. (**A**) Antigen-specific IgG level (ng/mL) at D21 and BD7. (B-D) Frequency (%) and cell number determined by flow cytometry of draining lymph node cells at day seven post-boost. (**B**) Absolute cell numbers of inguinal lymph nodes. (**C**) Frequency and cell number of GC B cells (GL7^+^CD95^+^ B cells). FACS plots were priorly gated on the total B cell (CD19^+^CD3^-^) population. (**D**) Frequency and cell number of T_FH_ cells (PD-1^+^CXCR5^+^ T cells). Flow cytometry plots were pre-gated on total T cell (CD19+CD3−) population. * *p* < 0.05, ** *p* < 0.01, *** *p* < 0.001. Data are representative of two independent experiments and are described as median, 25th, and 75th percentiles and a range.

To evaluate the antigen-specific antibody response induced by plant-produced G4NA, we measured the G4NA-specific total IgGs in mice immunized with G4NA alone or with G4NA and Addavax (AV), which is an MF59-like oil-in-water-emulsion adjuvant, at day 21 (D21, 3 weeks post-priming) and day 7 post-boost (BD7) (Figure 6A). As a result, vaccination with G4NA alone could trigger a modest G4NA-specific IgG response at BD7 (36.28 (6.097–139.8) ng/mL) compared to that of Mock group (2.894 (2.084–6.877) ng/mL), but not detectable at D21 (Mock, 4.090 (1.993–4.306); G4NA, 4.121 (3.688–6.149) ng/mL) (Figure 6A). This indicates that G4NA alone can elicit an antibody response after the boost immunization. To examine if a vaccine adjuvant can enhance the G4NA-specific IgG response, AV was supplemented to the G4NA antigen inoculum. AV substantially potentiated the G4NA-specific antibody production both at D21 and BD7 (G4NA+AV: D21, 901.1 (431.6–5031) ng/mL; BD7, 194,082 (181,013–298,962) ng/mL) (Figure 6A). The boost vaccination at D21 increased IgG production by 8.8-fold in the G4NA alone group and 215-fold in the G4NA+AV group as compared with the medians. Here, we tested 2020/21 Vaxigrip Tetra, a licensed inactivated seasonal influenza vaccine containing the representative antigen, hemagglutinin, as a control influenza vaccine. As expected, the Vaxigrip Tetra-immunized mice did not show any NA-specific antibody response, although the HA-specific antibody was effectively generated. This is understandable because Vaxigrip Tetra is formulated based on the content of HA antigen (15 μg HA of each strain per dose), similar to other universal influenza vaccine formulations [39].

Germinal center (GC) formation and helper T cell response in the secondary lymphoid organ are crucial for strong induction of a T-dependent antibody response [40]. During the germinal center reaction, antigen-specific B cells undergo massive expansion, affinity maturation, and antibody class switching, which requires critical help from follicular helper T (T_FH_) cells. There was a trend of incremental total cellularity in the BD7 draining lymph nodes of all vaccinated groups, though only G4NA+AV showed statistical significance (Figure 6B). Consistent with the G4NA-specific antibody data, the G4NA-vaccinated group of mice displayed slightly increased frequencies and numbers of GC B cells and follicular helper T cells (T_FH_ cells) as compared to the Mock group, suggesting that the G4NA antigen is a potentially good candidate that may require a vaccine adjuvant for more potent immunogenicity (Figure 6C,D). Indeed, in the G4NA+AV group, frequencies and cell numbers of both GC B cells and T_FH_ cells were significantly more enhanced in the dLNs than in the Mock or G4NA groups (Figure 6C,D). In contrast to the NA-specific antibody response, a vaccine-triggered increase of GC B cells and T_FH_ cells could be detected in Vaxigrip Tetra-immunized mice. Based on these results, we suggest that G4NA induces a G4NA-specific antibody response and germinal center reaction, which can be enhanced when administered with an emulsion adjuvant, AV.

### 2.6. G4NA Elicits NA-Specific CD4 T Cell Responses

**Figure 7:**
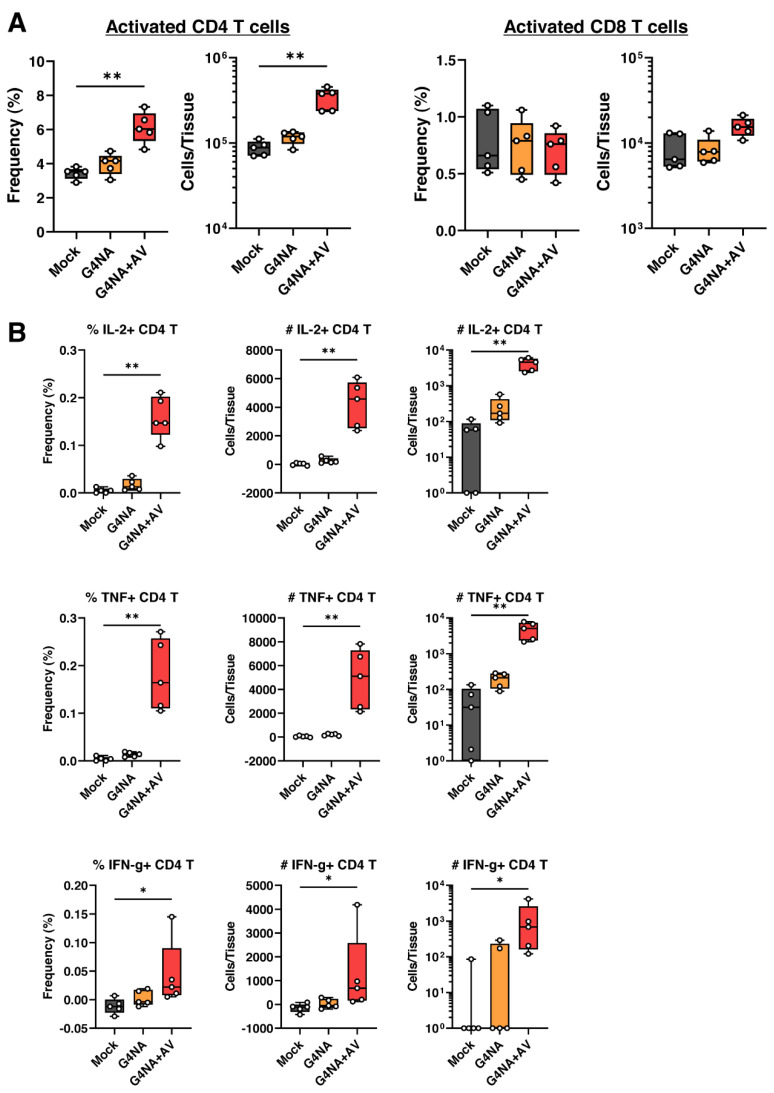
**G4NA promotes CD4 T cell responses.** (**A**) Frequencies (%) and cell numbers of activated CD4 T cells and activated CD8 T cells at day seven post-boost after vaccination with G4NA alone or with G4NA plus AV. (**B**) Frequencies (%) of TNF^+^/IL-2^+^/IFN-γ^+^ CD4 T cells in total CD4 T cells and total cell numbers (#) of each population. * *p* < 0.05, ** *p* < 0.01. Data are displayed as box plots showing median, 25th and 75th, percentiles, and the range.

We sought to examine if T cell response was triggered by G4NA immunization, in addition to the aforementioned T_FH_ cell response. The flow cytometry data of dLN cells from the immunized mice at BD7 indicates the increase of activated CD4 T cell responses after the secondary immunization in vivo (Figure 7A). The frequencies and numbers of activated CD4 (CD44^hi^CD62L^lo^) T cells were significantly increased in the G4NA+AV group compared to the Mock and non-adjuvanted vaccine groups. Contrastingly, there was no significant increase of activated CD8 (CD44^hi^CD62L^lo^) T cells either in G4NA or G4NA+AV (Figure 7A). The expansion of CD4 T cells after vaccination is important since the CD4 T cell responses are closely related to the vaccine-elicited antibody production and regulation of most immune responses [41,42]. Previous studies have reported significant HA-specific but inefficient NA-specific CD4 T cell responses after influenza vaccination [43]. However, recombinant G4NA in this study could induce significant CD4 T cell responses, implying that the antigen is capable of targeting CD4 T cells. On the other hand, CD8 T cell response, which was absent in the G4NA-vaccinated groups, is a means for the direct elimination of infected cells and is not effectively induced by conventional influenza vaccination [44]. These results imply that the in vivo administration of G4NA induces CD4 T cell responses, which is essential for the overall immunogenicity of the influenza vaccine.

To understand the aspect of antigen-specific T cell responses induced by the G4NA antigen, we performed ex vivo re-stimulation of T cells from dLNs with G4NA protein (Figure 7B). In response to the G4NA antigenic re-stimulation, CD4 T cells from the G4NA alone group induced slightly increased TNF expression. Strikingly, the G4NA+AV group showed a significant elevation in the percentages of IL-2-, TNF-, and IFN-γ-producing CD4 T cells. For the CD8 T cells, there was no significant changes in the percentage of IL-2/TNF/IFN-γ-producing cells after G4NA re-stimulation (Supplementary Figure S1). This is expected, as protein subunit antigens are not usually strong enough antigens to induce T cell responses in the absence of an immuno-stimulator. We could detect decent amounts of cytokine-producing CD4 T cells in response to G4NA antigen with an emulsion adjuvant, implying that G4NA harbors epitopes for CD4 T cells and potent B cell epitopes. Moreover, it is difficult to mount a potent CD8 T cell response with protein antigens unless the vaccine is formulated with special adjuvants that can induce an effective CD8 T cell response.

## 3. Materials and Methods

### 3.1. Plant Materials and Growth Conditions

*N. benthamiana* plants (NCBI:txid4100) were grown in a greenhouse at 23–24 °C and 40–65% relative humidity with a 16-h light/8-h dark cycle. The leaves of 6–7-week-old plants were used for agro-infiltration.

### 3.2. Plasmid DNA Construction

The *G4NA* sequence (NCBI, MN416747) was obtained using gene synthesis (Bioneer corp., Daejeon, Korea). The *NaeI* restriction site and a GGA sequence that codes for Gly were added at the 5′ end, and the sequences which code for the HA tag, HDEL ER retention signal, TAA stop codon, and *XhoI* restriction site were added at the 3′ end. In this study, to increase the solubility of G4NA, the sequence encompassing the N-terminal hydrophobic transmembrane domain (amino acids 1-34 in G4NA primary structure) was deleted. To generate the construct *BiP-M-CBM3-bdSUMO-G4NA-HDEL* (*MCS-G4NA*), the synthesized *G4NA* sequence was digested with *NaeI* and *Xho1* restriction endonucleases and inserted into the pUC-based vector digested with the same restriction endonucleases. The pUC-based vector used in this study contained the sequences that code for the BiP signal sequence, M-domain of the human receptor-type tyrosine-protein phosphatase C, family 3 cellulose-binding module (CBM3), bdSUMO, and HSP transcriptional terminator [45]. The resulting plasmid was digested with *XbaI* and *EcoRI*, and the digested fragment, which contains the sequences corresponding to the BiP-M-CBM3-bdSUMO-G4-NA-HDEL-(stop codon)-HSP terminator, was ligated into the pCambia 1300 plant expression vector digested with the same restriction endonucleases [17,46].

### 3.3. Agro-Infiltration of MCS-G4NA into the N. benthamiana Leaves

The construct *MCS-G4NA* was transformed into *Agrobacterium tumefaciens* (EHA105). *A. tumefaciens*, which is transformed with an *MCS-G4NA* construct, was introduced into *N. benthamiana* leaves via syringe infiltration as described previously [18,33]. In every agro-infiltration experiment, *A. tumefaciens* harboring p38, which is derived from *Turnip crinkle virus* and encodes a suppressor of host gene-silencing, was co-infiltrated.

### 3.4. Purification of MCS-G4NA from the N. benthamiana Leaves

A total of 20 g (fresh weight) of leaves, harvested at 3, 5, and 7 days after agro-infiltration, were frozen and ground in liquid nitrogen. Total protein extracts were prepared using 60 mL of protein extraction buffer (50 mM Tris-HCl pH 7.5, 150 mM NaCl, 1 mM DTT, 1 % [*v*/*v*] Triton X-100, and 1 X EDTA-free protease inhibitor cocktail (Roche, Switzerland). After incubation at 4 °C for 15 min, the total proteins were filtered through Miracloth (Merck Millipore, Burlington, MA, USA).

Subsequently, 100 µL of protein extracts were collected as the total (T) fraction. The remaining extracts were centrifuged at 19,400× *g* for 15 min at 4 °C, and 100 µL of the supernatant fraction was collected as the soluble (S) fraction. The pellets were resuspended in 60 mL of protein extraction buffer, and 100 µL of the sample was collected as the pellet (P) fraction. The soluble (S) fraction after centrifugation was used for the purification of MCS-G4NA with microcrystalline cellulose (MCC) beads (Sigma-Aldrich, St. Louis, MO, USA; CAS Number 9004-34-6) as described previously [18,33]. After purification of MCS-G4NA with MCC beads, the N-terminal upstream domains, which include CBM3 and bdSUMO, were removed by His-bdSENP1 protease, which was expressed in *E. coli* BL21 (DE3) pLysS [33]. Briefly, the MCS-G4NA immobilized on MCC beads was incubated with 10 µg of His-bdSENP1 in 10 mL reaction buffer (25 mM Tris-HCl buffer, pH 7.5, 0.1% NP-40, 250 mM NaCl and 1 mM DTT) for 6 h at 4 °C. The purified G4NA protein was concentrated using Amicon Ultra-0.5 Centrifugal Filter Unit (Cat# UFC501024, Merck KGaA, Darmstadt, Germany) and subjected to dialysis with phosphate-buffered saline (PBS). The concentration of G4NA was measured using bicinchoninic acid assay kit (Abbkine, cat no: KTD 3001, Wuhan, China).

### 3.5. Blue Native-Polyacrylamide Gel Electrophoresis (BN-PAGE)

BN-PAGE was performed for separation of total soluble leaf extracts under nondenaturing conditions using the NativePAGE Novex gel system (Invitrogen, MA, USA) with precast 4 to 16% acrylamide Bis-Tris gels (Invitrogen). After BN-PAGE, the gel was transferred onto a polyvinylidene fluoride membrane, followed by western blotting with anti-HA antibody (1:1000 dilution) (Roche, Catalog number: 11867423001, Basel, Switzerland).

### 3.6. Western Blot Analysis

The protein samples were separated by SDS-PAGE using 10 % acrylamide gel and transferred onto a polyvinylidene fluoride membrane. The membrane was incubated with 1 × TBS (Tris-buffered saline with 0.1 % (*v*/*v*) Tween 20) containing 6% (*w*/*v*) skim milk for 30 min. Then, the membrane was incubated with an anti-HA antibody (1:1000 dilution) at 4 °C overnight. After washing with 1 × TBS-T three times, the membrane was incubated with an anti-rabbit secondary antibody conjugated with horseradish peroxidase (1:5000 dilution) (Cell signaling Technology, cat no: 7074S) at 4 °C for 4 h. After washing with 1 × TBS-T three times, the membrane was immersed in ECL reagents (Thermo Fisher Scientific Inc., Waltham, MA, USA), and the chemiluminescence images were captured using the ChemiDoc^TM^ XRS+ imaging system (Bio-Rad Laboratories, Inc., Hercules, CA, USA).

### 3.7. Mice and Immunization

Six-week-old female Balb/c mice were purchased from Orient Bio Inc., Seongnam, Korea. Mice were immunized subcutaneously at the base of the tail with G4NA (10 μg per mouse) alone, G4NA plus Addavax™ (AV, 50 μL per mouse) (Invivogen), or Vaxigrip Tetra^®^ (80 μL per mouse) (Sanofi, Paris, France). Five mice per group were used in two independent experiments. Non-immunized mice were used as negative controls, and mice immunized with Vaxigrip Tetra^®^ (Sanofi) were used as positive controls. On day 21 post-immunization, mice were immunized with the same dose. We collected blood samples by submandibular (facial) bleeding on day 21 after the first immunization, and via cardiac puncture after euthanasia on day 7 post-boosting. Mice were maintained under specific pathogen-free conditions. Terminal anesthesia was conducted via carbon dioxide inhalation. The animal study was reviewed and approved by the Institutional Animal Care and Use Committee (IACUC) of Institut Pasteur, Korea (IACUC approval no. IPK-20012-1).

### 3.8. Antibody ELISA

Serum samples collected on day 21 after the first immunization and day 7 post-boosting were used for antibody ELISA. For serum separation, we clotted the blood samples for 1 h at 4 °C and centrifuged at 10,000× *g* at 4 °C for 10 min on the day of collection. Corning-EIA/RIA high-binding 96-well plates were coated with 50 μL of 2 μg/mL G4NA or 2 μg/mL goat anti-mouse immunoglobulin G (IgG) (Southern Biotech, Birmingham, AL, USA) by incubation at 4 °C overnight. Plates were washed twice with PBS with 0.5% Tween-20 and blocked with 200 μL of 1% blotting grade blocker (Bio-rad). Mouse serum samples were serially diluted (1:100 to 1:6400, 4-fold serial dilution) in blocking solution. The diluted samples were transferred to each well (50 μL/well) and incubated on the blocked plates at room temperature for two hours. Mouse IgG (50 μL/well) (Southern Biotech, Birmingham, AL, USA) was used as a standard after a 2-fold serial dilution from 100 to 1.56 ng/mL. The plates were washed twice and incubated with horseradish peroxidase (HRP)-conjugated secondary antibodies (anti-mouse IgG, 50 μL/well, 1:5000 dilution in blocking solution) (Southern Biotech, Birmingham, AL, USA) in the dark at room temperature for 1.5 h. After wash, HRP activity was developed using Tetramethylbenzidine (TMB) solution (100 μL/well, OptEIA reagent set, BD Biosciences, Franklin Lakes, NJ, USA) and stop solution (50 μL/well, 0.5 M hydrochloride). Optical density at 450 nm was detected using a spectorophotometer (Victor III, PerkinElmer, MA, USA) with correction at 595 nm.

### 3.9. Flow Cytometry

Draining lymph nodes (dLNs) were harvested from immunized mice in tissue harvest buffer (RPMI 1640 (Welgene) with 1% fetal bovine serum (FBS) and 1% of 1M N-2-hydroxyethylpiperazine-N-2-ethane sulfonic acid, HEPES). Cells from the dLN were stained with fluorochrome-labeled antibodies. The following antibodies were obtained from BioLegend: CD3 (17A2), CD44 (IM7), CD62L (MEL-14), IFN-γ (XMG1.2), IL-2 (JES6-5H4), TNF (MP6-XT22), CD19 (6D5), and GL7 (GL7). The following antibodies were purchased from BD Biosciences, NJ, USA: CD4 (RM4-5), CD8 (53-6.7), CD279 (PD-1; J43), CD185 (CXCR5; 2G8), and CD95 (Jo2). Zombie Aqua™ (BioLegend) was used to detect live/dead status. Fc block (BD Biosciences) was added during the cell staining. For intracellular staining, a Cytofix/Cytoperm solution kit (BD Biosciences) was used for the fixation and permeabilization of cells and antibody dilutions.

### 3.10. Ex Vivo T Cell re-Stimulation

dLN cells harvested from immunized mice were aliquoted 1 × 10^6^ cells/well in 96-well round-bottom cell culture plate (SPL Life Sciences, Pocheon-si, Korea). The cells were then stimulated with 1 μg/mL G4NA in complete RPMI (RPMI 1640 supplemented with 10% FBS and 1% of 1M HEPES) at 37 °C in a humidified 5% CO2 incubator for 16 h. A cell activation cocktail (phorbol 12-myristate-13-acetate and ionomycin, BioLegend) was used as a positive control. After the incubation, GolgiPlug (brefeldin A, BD Biosciences) and GolgiStop (Monensin, BD Biosciences) were added and incubated for four hours. Cells were then stained for flow cytometry analysis.

### 3.11. Statistical Analysis

Data are displayed as box plots of quantiles (median and 25th and 75th percentiles) and whiskers indicating a range. Statistical significance was determined by Kruskal–Wallis test with multiple comparisons by Dunn’s multiple comparisons test (ns, not significant; * *p* < 0.05; ** *p* < 0.01; *** *p* < 0.001). For analysis of cell re-stimulation data (Figure 7B), we compared the differences between the two groups by the Mann–Whitney *U* test (ns, not significant; * *p* < 0.05; ** *p* < 0.01). All statistical analyses were performed using Prism software version 9 (Graphpad Software, Inc., San Diego, CA, USA).

## 4. Conclusions

In this study, we provide compelling evidence that G4NA expressed in *N. benthamiana* leaves has great potential as a vaccine antigen for G4 EA H1N1. G4NA produced in plants was not only highly soluble and stable, but also assembled as a tetramer, which is critical for the efficacy of NA-based influenza vaccines. Moreover, it could effectively induce antigen-specific antibody responses, which is a critical feature for the protective immunity of vaccines. In the future, it will be necessary to further validate the G4NA vaccine in animal models with a live G4 EA H1N1 virus to investigate whether G4NA antigen has protective efficacy in vivo.

## Data Availability

Not applicable.

## Supplementary Materials

The following supporting information can be downloaded at: https://www.mdpi.com/article/10.3390/plants11212984/s1, Figure S1: **G4NA does not promote significant CD8 T cell responses.** Frequencies (%) of TNF^+^/IL-2^+^/IFN-γ^+^ CD4 T cells in total CD8 T cells and total cell numbers of each population. Data are displayed as box plots showing the median, 25th, and 75th percentiles, and the range.
