# Supplementary material for: In planta Production and Validation of Neuraminidase Derived from Genotype 4 Reassortant Eurasian Avian-like H1N1 Virus as a Vaccine Candidate
Source: Plants (Basel). 2022 Nov 4;11(21):2984. doi: 10.3390/plants11212984 (PMC9655071; doi:10.3390/plants11212984)
Supplement: Supplementary file 1 [file plants-11-02984-s001.zip › plants-2009223-supplementary.pdf]

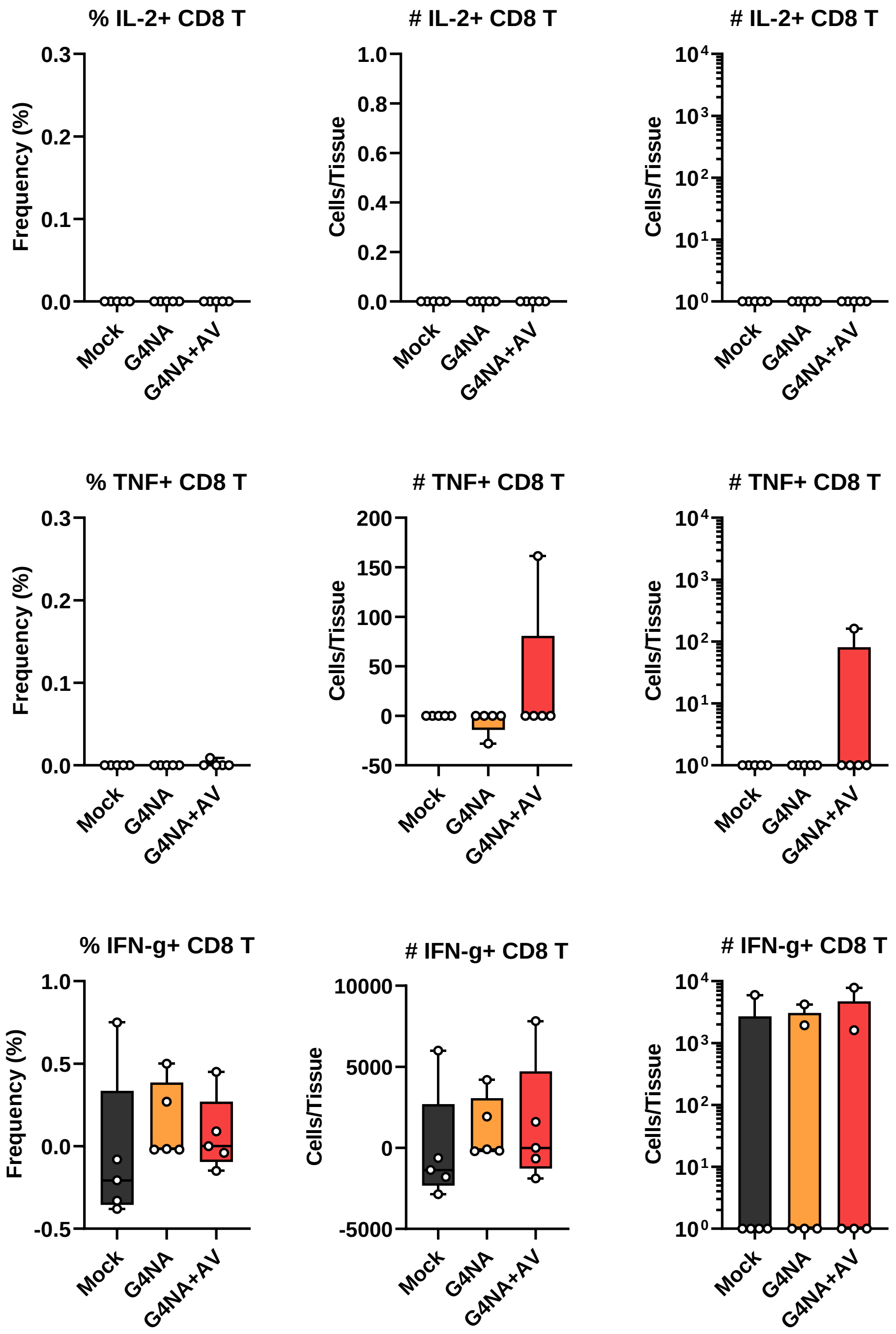

Supplementary Figure S1. G4NA does not promote significant CD8 T cell responses.

Frequencies (%) of TNF^+ / $IL\text{-}2^+$ / $IFN\text{-}\gamma^+$ CD4 T cells in total CD8 T cells and total cell numbers of each population. Data are displayed as box plots showing the median, 25th, and 75th percentiles, and the range.
